# Effects of treatment status and life quality on anxiety in MMT patients

**DOI:** 10.1186/s13011-021-00343-4

**Published:** 2021-01-13

**Authors:** Penghui Cao, Zhaohua Zhang, Jun Zhong, Shichao Xu, Qiaofang Huang, Ni Fan

**Affiliations:** The Affiliated Brain Hospital of Guangzhou Medical University (Guangzhou Huiai Hospital), 36 Mingxin Road, Liwan District, Guangzhou, 510370 Guangdong Province China

**Keywords:** Methadone maintenance treatment, Anxiety, Quality of life

## Abstract

**Background:**

Anxiety, an important factor that affects the therapeutic effect and preservation rate of methadone maintenance treatment, has a high prevalence among MMT patients. This study aims to investigate the effects of treatment status and life quality on anxiety in MMT patients.

**Methods:**

One hundred and Seventy-seven methadone maintenance treatment users in Guangzhou, China were evaluated. The socio-demographic, duration and MMT-related characteristics were documented. Anxiety level and quality of life were evaluated by Beck Anxiety inventory (BAI) and the Quality of Life-Drug Addiction (QOL-DA) respectively. The correlation between different factors and BAI score was also analyzed.

**Results:**

The BAI total score and the QOL-DA score were 7.1±8.2, 163.5±21.4 respectively. 30.5% of the subjects showed mild to severe anxiety. Treatment interruption and QOL-DA score had strong correlations with the score of BAI, with correlation coefficients of 0.17 and − 0.08 respectively.

**Conclusions:**

Anxiety symptoms were commonly presented in MMT patients. Treatment interruption and quality of life are two major factors affecting anxiety of MMT patients.

## Background

Opioid abuse remains a global problem, with implications for social security and the spread of diseases such as AIDS. There were around 53 million people worldwide had used opioids in 2018, and around 29 million people of them had used opiates such as heroin and opium [1]. Methadone is a synthetic μ-opioid receptor agonist that has similar effect to heroin [2]. Methadone maintenance treatment (MMT), a substitutive therapy, has been proved helpful in reducing opioid abuse, drug-related risk behaviors, the infection rate of AIDS and crimes [3–5], as well as improving patiIt is noteworthy that some researches indicated thatent’s overall wellbeing [6]. China started methadone maintenance treatment programs in 2004 and gradually promoted it across the country. By 2019, there were over 680 methadone therapy centers in China, serving about 300,000 MMT patients [7]. Currently, there are 63 MMT centers in Guangdong Province, and 12 of them are located in Guangzhou, the capital of Guangdong province.

Anxiety, is one of the major continued symptoms that a significant proportion of patients are suffering. It was reported that over 18% among MMT patients has anxiety symptom [8–11, 12]. It is noteworthy that some researches indicated that mental problems (including anxiety) were associated with preservation rate [8, 13, 14]. The preservation rate of MMT is a key point to reduce the risk of withdrawal symptoms, drug relapse and overdose due to decreased tolerance [15, 16]. Moreover, anxiety increase the risk of injection practices, HIV infection and criminal behaviors among MMT patients [10]. In addition, the high suicide rate of MMT patients has a strong correlation with anxiety [17]. Exploring anxiety and its influencing factors in MMT patients and give effective and positive interventions would be conducive to reducing a series of adverse risks such as relapse rate and suicide rate.

Previous studies indicated that education level, employment status [8], coincident drug use [18] and benzodiazepine use [19] are the factors that influence the anxiety in MMT patients. Low education, unemployment and positive urine drug test results and benzodiazepine use were the risks for anxiety among MMT patients [8, 18, 19]. However, there is still a lack of studies to comprehensively analyze the effects of MMT patients’ methadone treatment and life status on their anxiety.

This study focuses on the effects of treatment status and quality of life which gives a good assessment of the patient’s condition on anxiety in MMT patients. And we try to evaluate the different concerns more comprehensively. In the treatment status, we were concerned not only with the patient’s current treatment dose, but also with the cumulative duration of methadone treatment (the total number of months of methadone treatment from inception to evaluation) and whether the treatment had been discontinued. In the quality of life assessment, in order to make the results more reliable, the QOL-DA, a scale specifically designed for drug-addition patients, was also used in this study to assess patients’ life quality. Moreover, we focus not only on the total score of QOL-DA, but also on its four dimensions, striving to obtain more useful information from different dimensions.

## Methods

### Sample and data collection

This study was conducted from May to December 2019 in the MMT clinic of the Affiliated Brain Hospital of Guangzhou Medical University in Guangzhou, Guangdong Province. This clinic have provided treatment services for over 200 registered MMT patients in Guangzhou.

MMT patients were conveniently recruited if they were: (1) aged at least 18 years old; (2) received methadone treatment for at least 12 months; (3) accepted to participate and signed informed consent; (4) did not have severe cognitive impairments that might affect the communication between subjects and researchers. Among 210 MMT patients interviewed, 177 of them accepted to participate (response rate of 84.3%). All participants signed informed consent. All participants were involved in face-to-face interviews with researchers. Measures.

### Socio-demographic, cumulative duration of heroin use and MMT-related characteristics

Background data of each participant were recorded, including gender, age, years of education, living situation, marital status, drinking status and smoking status. Cumulative duration of heroin use was measured in months. Characteristics related to Methadone maintenance treatment was also recorded, including cumulative duration of methadone treatment (the total number of months of methadone treatment from inception to evaluation), current therapeutic does (the current daily dose of methadone, mg/d) and whether treatment had ever been interrupted.

### Standardized scales

The Beck Anxiety Inventory, a brief measure of anxiety with a focus on somatic symptoms of anxiety, was used to assess anxiety in MMT patients. The total score ranges from 0 to 63.0–9, normal/minimal level; 10–18, mild anxiety; 19–29, moderate anxiety; and 30–63, severe anxiety [20, 21]. The BAI was found to have excellent internal consistency (Cronbach α=0.92) [21].

The Quality of Life-Drug Addiction (QOL-DA) was used in this study to assess participants’ life quality. QOL-DA is a valid, reliable and responsive instrument for measuring the quality of life of opioid dependent individuals [22]. The QOL-DA was developed by Chong-hua Wan in 1997 for drug-dependent patients in China. The QOL-DA consists of 40 items that measure four dimensions include physiology, psychology, society and symptoms [22]. The reliability of the overall items, physiology, psychology, symptoms and society was 0.866, 0.795, 0.826, 0.914 and 0.714 [22, 23]. The higher score the subjects get, the better the quality of life they have.

### Statistical analysis

SPSS version 19.0 was used to analyze data. T-test (for data with continuous distribution) and Chi-square test were used to detect the differences of variables between two durations of anxiety: no anxiety and anxiety. A *p*-value <0.05(two-tails)was used to identify statistical significance. Pearson correlation and Spearman rank correlation were used to describe the association between different factors and BAI score.

## Results

### Characteristics of MMT patients

Socio-demographic characteristics, cumulative duration of heroin use, MMT-related characteristics, BAI score, QOL-DA score and the score of its four dimensions are presented in Table 1. Altogether there were 177 methadone maintenance treatment users enrolled in the study of which 155 were male (87.6%) and 22 were female (7.5%). The average age was 51.8±5.7 (Mean±SD). The average cumulative duration of heroin use was 261.4±82.6 months (Mean±SD) and the cumulative duration of MMT was 100.9±60.7 months (Mean±SD). The average dose of current methadone treatment was 37.3±22.1 mg (Mean±SD). There were 72 subjects (40.7%) had ever interrupted methadone treatment. The average BAI score and the average QOL-DA score were 7.1±8.2 and 163.5±21.4 respectively.

**Table 1:** Characteristics of MMT users

	MMT users (*N*=177)
Gender (males/females)	155(87.6%)/22(12.4%)
Age	51.8±5.7
Years of education	
≤9	131(74.0%)
10–12	44(24.9%)
≥13	2(1.1%)
Marital status (single/married)	98(55.4%)/79(44.6%)
Living situation (alone/with partners)	57(32.2%)/120(67.8%)
Drinking status (yes/no)	58(32.8%)/119(67.2%)
Smoking status (yes/no)	174(98.3%)/3(1.7%)
Cumulative duration of heroin use (month)	261.4±82.6
Cumulative duration of MMT (month)	100.9±60.7
Current methadone therapeutic dose (mg)	37.3±22.1
MMT Interruption (yes/no)	72(40.7%)/105(59.3%)
BAI score	7.1±8.2
QOL-DA total score	163.5±21.4
Physiology Score	33.3±6.5
Psychology Score	39.6±6.8
Society Score	39.2±6.9
Symptoms Score	48.3±7.2

Data are the amount of cases (percentage). Data about age, cumulative duration of heroin use, cumulative duration of MMT, current methadone dose and QOL-DA score are the mean±standard deviation

**Fig. 1:**
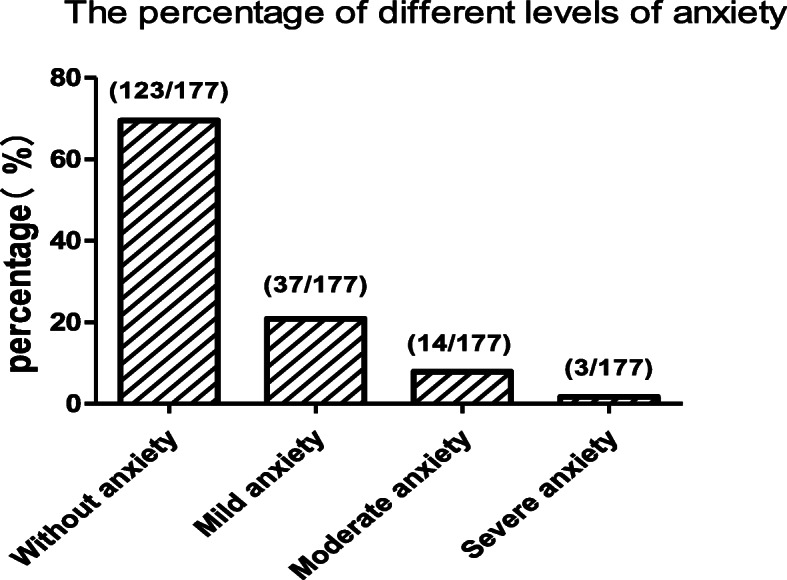
MMT users’ evaluation by BAI. The percentages of subjects clarified by the cutoffs on BAI

The percentages of subjects clarified by BAI scale are presented in Fig. 1. For the measure of anxiety, 123(69.5%) subjects showed minimal level of anxiety (score 0–9), 37(20.9%) subjects showed mild anxiety (score 10–18), 14(7.9%) subjects showed moderate anxiety (score 19–29), 3(1.7%) subjects showed severe anxiety (score 30–63), for a total of 30.5% subjects had varying degrees of anxiety.

### Comparison between anxiety group and non-anxiety group

Subjects were divided into two groups according to BAI score: anxiety group (score 10–63) and non-anxiety group (score 0–9) [20, 21]. Fifty-four subjects (30.5%) were divided into anxiety group and 123 subjects (69.5%) were divided into non-anxiety group. The characteristics of the anxiety group and non-anxiety group were compared.

The comparison results of various characteristics between anxiety group and non-anxiety group are shown in Table 2. There was no statistical difference in gender, age, marital status, living situation, drinking status, smoking status, cumulative duration of heroin use and cumulative duration of treatment between anxiety group and non-anxiety group. Meanwhile, those suffered from anxiety were currently treated at higher doses(42.7±19.2 mg) compared to those did not suffer from anxiety (34.9±22.9 mg)(*p*<0.05, Cohen’s d=0.37). The proportion of those had ever interrupted treatment in anxiety group (55.6%) is higher than that in non-anxiety group (34.1%) (*p*<0.01). And the odds ratio of MMT interruption is 2.4, which indicated the effect size is large. The QOL-DA score the subjects in the anxiety group got was 143.13±22.3, which was lower than that (172.46±13.52) in the non-anxiety group. And the effect size is also very large (Cohen’s d=− 1.59). This result indicated that the quality of life of the anxiety group’s subjects was not as good as that of the non-anxiety group’s subjects.

**Table 2:** The comparison between anxiety group and non-anxiety group

	Anxiety group (*n*=54)	Non-anxiety group (*n*=123)	*p*-value
Gender (males/females)	45(83.3%)/9(16.7%)	110(89.4%)/13(10.6%)	0.26
Age	51.0±5.1	52.2±5.9	0.20
Years of education			
≤9	40(74.1%)	91(74.0%)	0.63
10–12	14(25.9%)	30(24.4%)	
≥13	–	2(1.6%)	
Marital status (single/married)	30(55.6%)/24(44.4%)	68(55.3%)/55(44.7%)	0.97
Living situation (alone/with partners)	21(38.9%)/33(61.1%)	36(29.3%)/87(70.7%)	0.23
Drinking status (yes/no)	16(29.6%)/38(70.4%)	42(34.1%)/81(65.9%)	0.56
Smoking status (yes/no)	54(100.0%)/−	120(97.6%)/3(2.7%)	0.25
Cumulative duration of heroin use (month)	253.9±76.3	264.7±85.3	0.43
Cumulative duration of MMT (month)	113.4±53.1	95.4±63.4	0.07
Current methadone therapeutic dose (mg)	42.7±19.2	34.9±22.9	0.03*
MMT Interruption (yes/no)	30(55.6%)/24(44.4%)	42(34.1%)/81(65.9%)	0.008**
QOL-DA total score	143.13±22.3	172.46±13.52	<0.01**

Data are the amount of cases (percentage). Data about age, cumulative duration of heroin use, cumulative duration of MMT, current methadone dose and QOL-DA score score are the mean±standard deviation

T-test, Chi-square test and Fisher’s exact test were used to detect the differences of variables between two durations of anxiety: no anxiety and anxiety.

* *p*<0.05

**p<0.01

### Correlation between different factors and BAI score

In order to explore the influence of therapy status and life quality on anxiety, this study analyzed the correlation between therapy status, QOL-DA score and BAI score. The correlation between BAI score and other personal conditions and the cumulative duration of heroin use were also explored.

Bivariate correlation analysis (Table 3) showed that the BAI score was negatively correlated with age, smoking status and QOL-DA score, while it was positively correlated with other factors (such as cumulative duration of heroin use, cumulative duration of MMT, MMT interruption, etc.). Among all the factors, the correlation between MMT interruption, QOL-DA score and BAI score were statistically significant (*p*<0.05), and the correlation coefficients are 0.17 and − 0.80 respectively. It means that MMT patients who had ever interrupted from methadone maintenance treatment may have higher risk of anxiety. Moreover, it can also be concluded that the lower QOL-DA score that subjects got, the higher BAI score they got. In other word, the worse life quality the subjects had, the more severe anxiety they suffered from.

**Table 3:** The correlation between different factors and BAI score

	BAI Score
Gender	0.12
Age ^&^	−0.11
Years of education	0.02
Marital status	0.01
Living situation	0.09
Drinking status	0.03
Smoking status	−0.16
Cumulative duration of heroin use (month)^&^	0.01
Cumulative duration of MMT (month) ^&^	0.06
Current methadone therapeutic dose ^&^	0.08
Change of methadone therapeutic dose	0.04
MMT Interruption	0.17*
QOL-DA Score^&^	−0.80**
Physiology Score ^&^	−0.69**
Psychology Score ^&^	−0.79**
Society Score ^&^	−0.60**
Symptoms Score ^&^	−0.72**

Values listed in the table were R^2^ for Spearman correlation. Physiology, psychology, society and symptoms are four scales of QOL-DA

^&^ R^2^ for Pearson correlation

^*^ *p*<0.05

^**^p<0.01

## Discussion

In this study, we found that 30.5% MMT patients had anxiety symptoms. We also found that the dosage of methadone in anxiety group was higher than that in non-anxiety group. Compared with the non-anxiety group, the anxiety group had a higher methadone treatment interruption rate. We also found that good life quality had positive effects on reducing the risk of anxiety.

We found that 30.5% MMT patients in this study suffered from anxiety. The proportion was lower than that shown by previous study conducted in Australia (67.7%) [9], but it was similar to that of another two studies conducted in China (23.0 and 33.6% respectively) [10, 11]. Although only 177 subjects were included in this study, the proportion of anxiety was approach to that of previous Chinese studies. This means that the results in this study may reflect the overall situation to a certain extent. Surprisingly, the anxiety proportion in this study was far lower than that in the previous Australian study. The discrepancy may due to the cumulative duration of methadone treatment of participants in studies. In a Chinese study [10], it was found that the longer the maintenance treatment lasted, the lower the prevalence of anxiety disorders among participants. The methadone treatment time for Australian participants is merely 1 week to 6 months, which is shorter than that in our study. It is not difficult to understand that the possibility and proportion of patients with anxiety symptoms would be relatively high when the drug use has not reached full dose and sufficient treatment. Therefore, it is possible that the duration of methadone maintenance treatment may be a crucial factor affecting the prevalence of anxiety among MMT patients. Full dose and sufficient treatment are advocated. Most of the subjects in this study were accepted in the MMT program more than 12 months and were in stable stage. In this situation, there were still 30.5% of the subjects with anxiety, which indicated that anxiety, as a missed symptom, has a high probability in MMT patients.

When we compared the differences of different factors between anxiety group and non-anxiety group, we found that the methadone therapeutic dose in anxiety group was higher than that in non-anxiety group. That was in contrast with the result of a previous survey conducted in China [8]. That study indicated that low therapeutic dose increases the risk of anxiety. Higher dose of methadone could reduce heroin craving, inhibit heroin’s euphoric effect, prevent withdrawal symptoms and stabilize the behavior of drug addicts. Therefore, full dose treatment is recommend. Moreover, the higher dose of methadone is also related to the MMT retention rate increase and the improvement in treatment effectiveness [24–27]. Several studies had suggested that MMT patients with mental problems or disorders might need higher therapeutic dose of methadone to get better treatment benefits [24, 25]. However, the dose of methadone varies from person to person. Therefore, doctors of MMT center prescribing adequate dose of methadone to patients, according to the patients’ personal situation, is an effective way to reduce the risk of anxiety in MMT patients. In this study, a significant number of subjects had been on methadone maintenance therapy for more than 2 y. Some of them try to gradually reduce their daily dose of methadone with the help of a doctor at a methadone treatment center, depending on how they feel about their condition. When MMT patients feel good about themselves, they take the initiative to communicate with their doctors and reduce their daily treatment dose under their doctor’s guidance. Therefore, it is easy to understand that subjects in the non-anxious group did not suffer from anxiety and had a better personal state, and a lower daily dose of therapy could help them maintain a good personal state. At the same time, the active self-reporting of MMT patients also helps doctors in methadone maintenance treatment centers to make timely adjustments to patients’ personal treatment plans. However, there are still some patients who only treat according to the doctor’s original plan, but do not actively express their discomfort, leading to a mismatch between the treatment dose and their personal conditions. This may also be the reason why a large proportion of patients are still experiencing anxiety. Therefore, in addition to the patient’s active statement of their condition, it is also necessary for methadone treatment centers to provide daily reassessment for MMT patients. However, in China, doctors of methadone treatment centers are overworked. They provide therapeutic services to dozens MMT patients daily, and rarely have enough time to conduct a detailed daily reassessment of every patient thy serve. Therefore, improving the current staffing of methadone treatment centers in China and increasing the number of trained professionals (such as trained psychological counselors) to conduct daily reassessment of MMT patients would not only improve the effectiveness of methadone maintenance treatment, but also reduce the risk of anxiety in MMT patients.

The percentage of subjects who had ever interrupted treatment in the anxiety group was higher than that in the non-anxiety group, and the effect size is large (OR=2.4). Moreover, the correlation analysis showed that MMT interruption was positively correlated with BAI score, although the correlation intensity was weak. Referring to the results of previous studies, it is undeniable that there is an inseparable relationship between treatment interruption and anxiety. During the interview, some of the patients said that the reason they stopped treatment was that they thought they could no longer rely on methadone maintenance treatment. They supposed that they had good self-control. However, contrary to their wishes, they began to have some bad feelings after they stopped the treatment. Even some of them are back to abusing opioids. In the end, they had to go back to the methadone treatment center and ask for help. This is another reminder of the importance of daily reassessment. It not only optimizes therapy regimens, but also makes MMT patients know more clearly about their personal condition when they are reassessed.

In this study, we also found that the overall life quality of the subjects in anxiety group was worse than that of the subjects in non-anxiety group, and the effect size is also large (Cohen’s d=− 1.59). This result was in line with a previous study which showed that psychiatric problems (included anxiety) were associated with lower quality of life [28]. The results of correlation analysis in our study also showed that there was a negative correlation between the DA-QOL score and BAI score, and the correlation coefficient is − 0.80 which suggesting a strong correlation. This result indicated that MMT patients with anxiety may have poorer life quality than those without anxiety. Moreover, it also indicated that the poorer life quality the MMT patients have, the higher level anxiety they may have. This result is consistent with the results of previous studies suggesting that there is a certain correlation between quality of life and emotional problems of MMT patients [29–31]. This study not only focused on the relationship between the overall evaluation of life quality and the level of anxiety, but also focused on the correlation between the four dimensions of the evaluation of life quality (physical condition, psychological state, social function and withdrawal symptoms) and the level of anxiety. That makes the evaluation more comprehensive. The results of this study were similar to the results of previous studies: all the four dimensions score had a strong negative correlation with the level of anxiety, which means that MMT patients had better physical status, better psychological status, better social support and fewer withdrawal, and had lower levels of anxiety. Previous studies had suggested that MMT patients’ good physical condition, good psychological state, sufficient social support and less withdrawal symptoms had positive effects on reducing the risk of anxiety [28, 29, 31]. Good physical condition and less withdrawal symptoms mean that MMT patients will bear less burden caused by physical problems, while good psychological state and good social function mean that MMT patients will bear less psychological burden. Therefore, as suggested by previous studies, improving MMT patients’ quality of life by improving their physical condition, psychological state, social function and reducing their withdrawal symptoms is an effective way to reduce the risk of anxiety [28, 29, 32].

From the result of the correlation between the four dimensions score and the level of anxiety, we can obtain some meaningful inspirations. In order to reduce the rate of drug abuse and some of the harms caused by drug abuse, the China government has opened more than 600 methadone treatment centers across the country and provided financial assistance to MMT patients to encourage them to actively and continuously receive methadone treatment. In addition, the community service centers provide a variety of counseling and reemployment services for MMT patients, which is of great help for MMT patients to return to society. However, there are still significant barriers to social reintegration of MMT patients. General readmission to substance abusers remains low, even if MMT patients have made up their minds and stick to detoxification treatment. As a result, MMT patients who are alienated from other people (including their relatives or friends) often suffer from loneliness and are more likely to have anxiety. Therefore, how to improve the general public’s understanding of MMT patients and reduce the resistance to MMT patients can be more conducive to the social reintegration of MMT patients and reduce the risk of anxiety.

Moreover, improvement in the three dimensions of physical, psychological and withdrawal symptoms requires more support from methadone treatment centers. The key to improving the physical state of patients and reducing withdrawal symptoms is to provide appropriate personalized treatment for MMT patients in treatment centers. As mentioned above, improving the staffing structure of treatment centers and providing professional and timely reassessment can make treatment more efficient and help reduce the risk of discomfort during MMT patients receive treatment, thus reducing the risk of anxiety. Furthermore, psychological support is also important. However, the majority of methadone treatment clinics and treatment centers in China only provide methadone medication and medication-related counseling for MMT patients, and a fraction of treatment centers had social workers in order to assist doctors. Psychological counseling and psychotherapy services are seldom parts of methadone maintenance treatment services in China [32, 33]. The necessary psychological counseling and some psychological treatment (such as cognitive behavioral therapy) may make patients better aware of their own psychological state changes and encourage them to actively improve their psychological state. Thus, providing necessary psychological counseling and treatment services for MMT patients is also one of the feasible ways to reduce the risk of anxiety for MMT patients. To improve the current situation of limited psychological service resources in China, to equip methadone treatment centers with professional psychotherapists and to provide psychological intervention services for MMT patients are all indispensable. This is also advocated by relevant research [32, 34].

### Limitation

This study is a cross-section study, without dynamic observation of the influencing factors. In addition, this study only divided the anxiety group and non-anxiety group among MMT patients for comparison, no further group comparisons were made between subjects with different levels of anxiety.

## Conclusions

A significant proportion of MMT patients suffer from anxiety. Treatment-related factors (e.g. interruption of treatment) and quality of life may directly or indirectly affect anxiety in MMT patients. Improving the staffing of methadone treatment centers, providing MMT patients with better personalized treatment plans, necessary psychological inquiry and psychological treatment services, and improving the acceptance of the general public to MT patients may be conducive to reducing the risk of anxiety in MMT patients.

## Data Availability

In order to protect the confidentiality and anonymity of participants, the data (transcripts) will not be shared.

## Abbreviations

MMT: Methadone Maintenance Treatment
BAI: Beck Anxiety Inventory
QOL-DA: Quality of Life-Drug Addiction
AIDS: Acquired Immune Deficiency Syndrome
HIV: Human Immunodeficiency Virus
